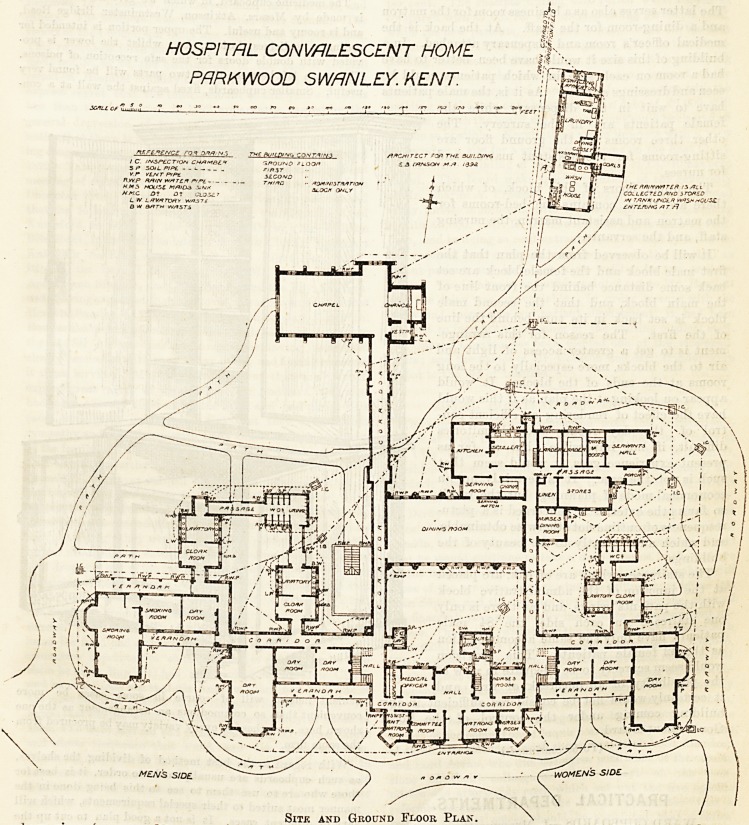# Hospital Convalescent Home, Parkwood, Swanley, Kent

**Published:** 1893-12-02

**Authors:** 


### The Institutional Workshop.

### HOSPITAL CONSTRUCTION.

### HOSPITAL CONVALESCENT HOME, PARKWOOD, SWANLEY, KENT.

The history of the foundation of this excellent institution, with some account of the building, has already been given (see page 336, Vol. XIV). We now publish plans of the ground and first floors, showing the details of the arrangements.

The site is about a mile and a half from Swanley Junction, and is on high ground, with extensive views both to the north and south.

The grounds, which were formerly attached to a house from which the name "Parkwood" is derived, and which is still standing, are extensive and beautifully wooded, and afford within themselves ample spacc for exercise and recreation for the patients.

The accommodation at present is for 84 male patients and 42 female patients, but the buildings have been planned with a view to a considerable increase of these numbers at a future time.

Consequent upon this inequality of the numbers the main administrative block does not occupy the exact centre of the buildings. It has on the east one block

Site and Ground Floor Plan.

for female patients, and on the west two blocks for male patients.

In the centre of the main administrative block is the principal entrance. Immediately opposite the vestibule is a large hall, in which is a bay window and a handsome stone chimney piece and dog grate. Entered from this hall are two rooms in front, being respectively the matron's sitting-room and the committee-room. The latter serves also as a business room for the matron and a dining-room for the staff. At the back is the medical officer's room and dispensary attached. In a building of this size it would have been better to have had a room on each side in which patients could be seen and dressings applied. As it is, the male patients have to wait in the dispensary while the female patients are in the surgery. The other three rooms on the ground floor are sitting-rooms for the assistant matron and for nurses.

The upper floors of this block, of which there are three, contains the bed-rooms for the matron and assistant matron, the nursing staff, and the servants.

It will be observed from the plan that the first male block and the female block are set back some distance behind the front line of the main block, and that the second male block is set back in its turn behind the line of the first. The reason for this arrangement is to get a greater access of light and air to the blocks, more especially to the long rooms at the ends of the blocks. It would appear on looking at the plan as if this would have the effect of rendering an efficient control of the day rooms and dormitories difficult, if not impossible; but so far as present experience goes, it would seem that such is not the case. It is certainly not an economical mode of planning, but the result so far as the exterior is concerned is a picturesqueness of outline not otherwise obtainable, and which adds greatly to the beauty of the buildings.

The staircases, which are of oak, are placed at the junction of the administrative block with the patients' wings, and as there is only one staircase on each side, the 84 male patients have only the same accommodation as the 42 female patients. This provision would seem scarcely to be adequate in view of the possibility of an outbreak of fire; and it certainly would not be considered sufficient in any building coming under the control of the Local Government Board.

(*To be continued.*)

## Figures and Tables

**Figure f1:**